# Clinical Features and Risk Factors of Oedematous *Mycobacterium ulcerans* Lesions in an Australian Population: Beware Cellulitis in an Endemic Area

**DOI:** 10.1371/journal.pntd.0002612

**Published:** 2014-01-02

**Authors:** Daniel P. O'Brien, N. Deborah Friedman, Anthony McDonald, Peter Callan, Andrew Hughes, Eugene Athan

**Affiliations:** 1 Department of Infectious Diseases, Barwon Health, Geelong, Australia; 2 Department of Medicine and Infectious Diseases, Royal Melbourne Hospital, University of Melbourne, Australia; 3 Manson Unit, Médecins Sans Frontières, London, United Kingdom; 4 Department of Plastic Surgery, Barwon Health, Geelong, Australia; Kwame Nkrumah University of Science and Technology (KNUST) School of Medical Sciences, Ghana

## Abstract

**Introduction:**

Oedematous lesions are a less common but more severe form of *Mycobacterium ulcerans* disease. Misdiagnosis as bacterial cellulitis can lead to delays in treatment. We report the first comprehensive descriptions of the clinical features and risk factors of patients with oedematous disease from the Bellarine Peninsula of south-eastern Victoria, Australia.

**Methods:**

Data on all confirmed *Mycobacterium ulcerans* cases managed at Barwon Health, Victoria, were collected from 1/1/1998–31/12/2012. A multivariate logistic regression model was used to assess associations with oedematous forms of *Mycobacterium ulcerans* disease.

**Results:**

Seventeen of 238 (7%) patients had oedematous *Mycobacterium ulcerans* lesions. Their median age was 70 years (IQR 17–82 years) and 71% were male. Twenty-one percent of lesions were WHO category one, 35% category two and 41% category three. 16 (94%) patients were initially diagnosed with cellulitis and received a median 14 days (IQR 9–17 days) of antibiotics and 65% required hospitalization prior to *Mycobacterium ulcerans* diagnosis. Fever was present in 50% and pain in 87% of patients. The WCC, neutrophil count and CRP were elevated in 54%, 62% and 75% of cases respectively. The median duration of antibiotic treatment was 84 days (IQR 67–96) and 94% of cases required surgical intervention. On multivariable analysis, there was an increased likelihood of a lesion being oedematous if on the hand (OR 85.62, 95% CI 13.69–535.70; P<0.001), elbow (OR 7.83, 95% CI 1.39–43.96; p<0.001) or ankle (OR 7.92, 95% CI 1.28–49.16; p<0.001), or if the patient had diabetes mellitus (OR 9.42, 95% CI 1.62–54.74; p = 0.02).

**Conclusions:**

In an Australian population, oedematous *Mycobacterium ulcerans* lesions present with similar symptoms, signs and investigation results to, and are commonly mistakenly diagnosed for, bacterial limb cellulitis. There is an increased likelihood of oedematous lesions affecting the hand, elbow or ankle, and in patients with diabetes.

## Author Summary

The oedematous form of Buruli ulcer, caused by *Mycobacterium ulcerans*, is less common representing 7% of cases over a 15 year period in a patient cohort from the Bellarine Peninsula of south-eastern Victoria, Australia. In this study, for oedematous Buruli ulcer cases, fever and pain were usually present and investigations showed leucocytosis, neutrophilia and a raised serum CRP. This is in contrast to other forms of Buruli ulcer which are classically painless and not associated with systemic symptoms. As a result oedematous cases were usually misdiagnosed and treated as bacterial cellulitis leading to delays in diagnosis, progression of disease, increased morbidity and increased complexity of treatment. Compared with non-oedematous forms of Buruli ulcer, we found that oedematous lesions were strongly associated with being located on the dorsum of the hand, the elbow and the ankle, and with patients who had diabetes mellitus. This study aims to increase the awareness of odematous Buruli ulcer disease, and improve the understanding of its clinical presentation and risk factors, to aid clinicians to diagnose and treat early *Mycobacterium ulcerans* infection when managing patients with cellulitis who have been exposed to areas endemic for Buruli ulcer.

## Introduction

Oedematous lesions are a form of non-ulcerative *Mycobacterium ulcerans* disease that can be rapidly progressive and cause significant tissue damage, even despite appropriate antibiotic treatment [1], [2]. They are a less common form of disease, reported to represent 0.7%–6% of cases in Benin, 3.3% of cases in northern Australia and 15% of lesions in Ghana [3]–[6]. Histopathology of lesions reveals an intact epidermis with wide contiguous coagulation necrosis and large numbers of acid fast bacilli (AFB) in the deep dermis, subcutaneous tissue, and fascia, with spread of AFB along fasical planes [7]. The cause of this disease form is not known, but it likely relates to a balance of host factors such as immune function and organism factors such as virulence and inoculum [7].

Cases of oedematous *M. ulcerans* infection can be misdiagnosed as bacterial cellulitis leading to delays in diagnosis, progression of disease, increased morbidity and increased complexity and cost of treatment [1], [2], [8]. Studies looking at the presenting clinical features and risk factors for oedematous lesions have not been performed, yet this information would be important to aid clinicians to diagnose these forms early and commence appropriate treatment. To address this information gap we have performed the first comprehensive descriptions of the clinical features and risk factors of patients presenting with oedematous forms of disease from a cohort of patients from the Bellarine Peninsula of south-eastern Victoria, Australia.

## Methods

### Ethics statement

This is an observational cohort study, approved by Barwon Health's Human Research and Ethics Committee. All previously gathered human medical data were analysed anonymously.

### Data collection

Data on all confirmed *M. ulcerans* cases managed at Barwon Health, Victoria, were collected prospectively from 1/1/1998–31/12/2012.

### Case definitions

An oedematous lesion was defined as a lesion at the time of initial presentation to the health-care provider that was manifest by diffuse, extensive, usually non-pitting swelling with ill-defined margins involving part or all of a limb or other part of the body [9]. A *M. ulcerans* case was defined as the presence of a lesion clinically suggestive of *M. ulcerans* plus any of (1) a culture of *M. ulcerans* from the lesion, (2) a positive IS2404 real-time PCR from a swab or biopsy of the lesion, or (3) histopathology of an excised lesion showing a necrotic granulomatous ulcer with the presence of acid-fast bacilli (AFB) consistent with acute *M. ulcerans* infection. Fever was defined as a temperature ≥38 degrees Celsius. Immune suppression was defined as current treatment with immunosuppressive medication or an active malignancy.

### Statistical analysis

Data was collected using Epi-Info 6 (CDC, Atlanta) and analysed using STATA 12 (StataCorp, Texas, USA). Proportions were compared using 2×2 tables and the Chi-squared test. Medians of non-parametric variables were compared using the Wilcoxon rank sum test.

A logistic regression model was used to assess associations of variables with oedematous forms of *M. ulcerans*. Unadjusted odds ratios were determined by performing univariable analyses. A multivariable analysis was performed including the variables sex and age *a priori* and all variables showing strong evidence of an association with oedematous lesions in the univariable analysis (assessed by p≤0.20). P-values were determined using the likelihood ratio test.

## Results

### Overall study population

**Figure 1:**
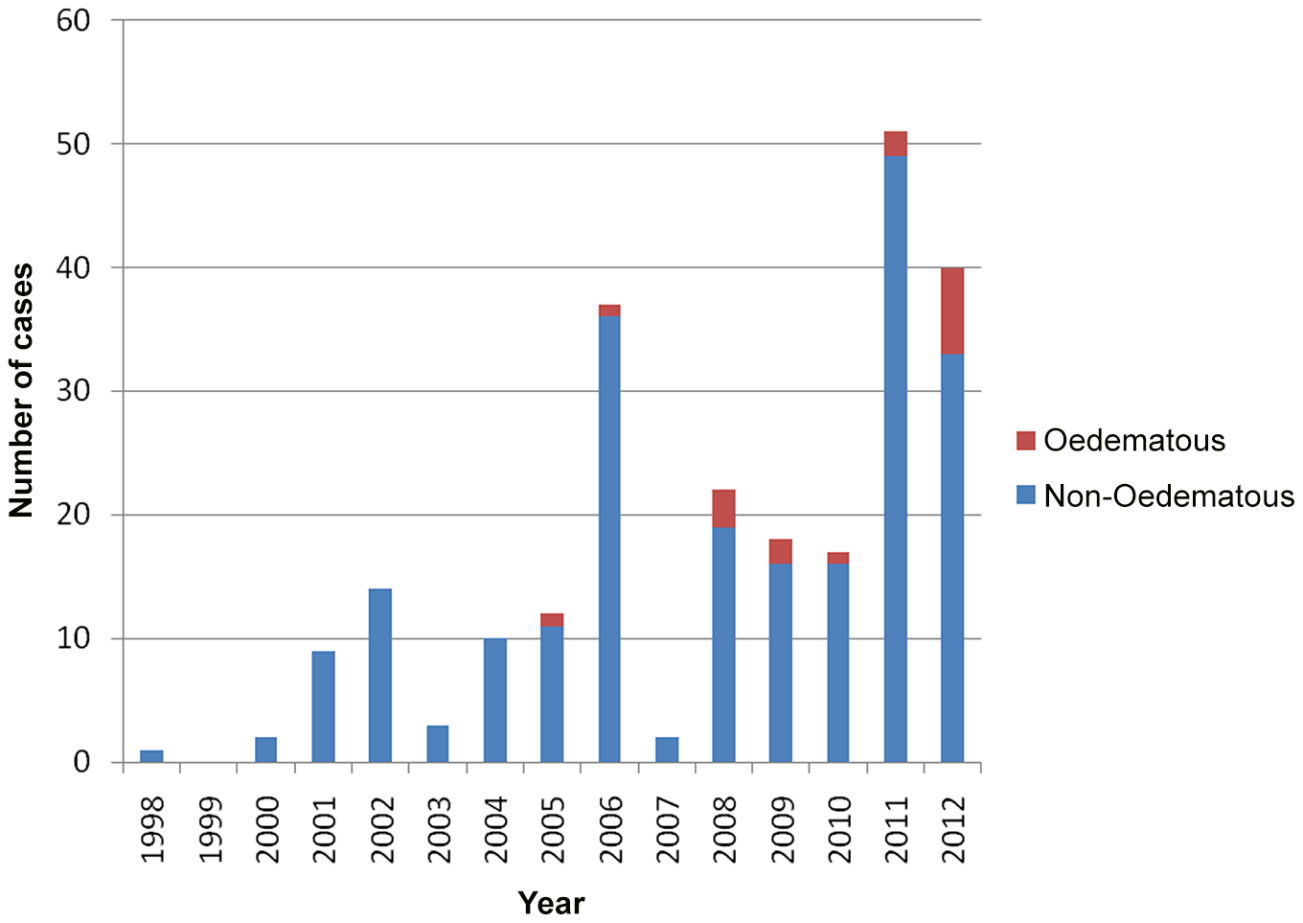
Odematous and non-odematous Buruli ulcer cases from the Bellarine Peninsula, Australia, managed at Barwon Health 1998–2012.

During the study period 240 patients with *M. ulcerans* were managed at our health service. Two patients did not have their type of lesion recorded and were excluded from further study. Seventeen of 238 (7%) patients with their type of lesion recorded had oedematous lesions. There was a trend to an increasing proportion of oedematous cases over the study period [0/29 (0%) cases 1998–2002, 2/61 (3%) cases 2003–2007, 15/148 (10%) cases 2008–2012; p = 0.09] (Figure 1).

### Patients with oedematous lesions

**Figure 2:**
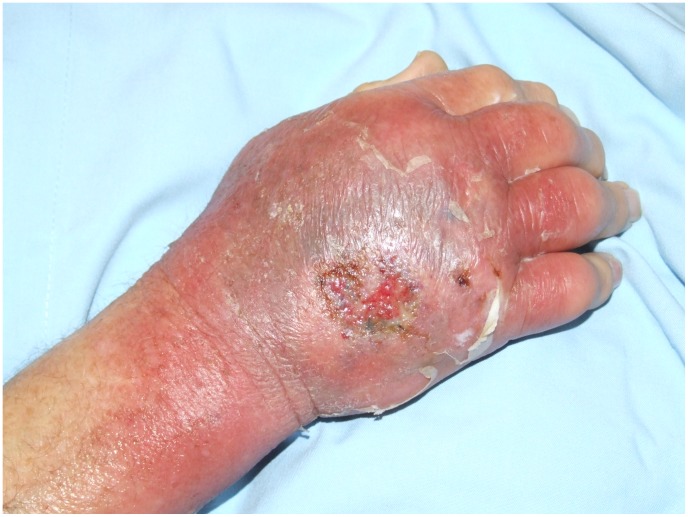
Oedematous *M. ulcerans* lesion dorsum of left hand.

**Figure 3:**
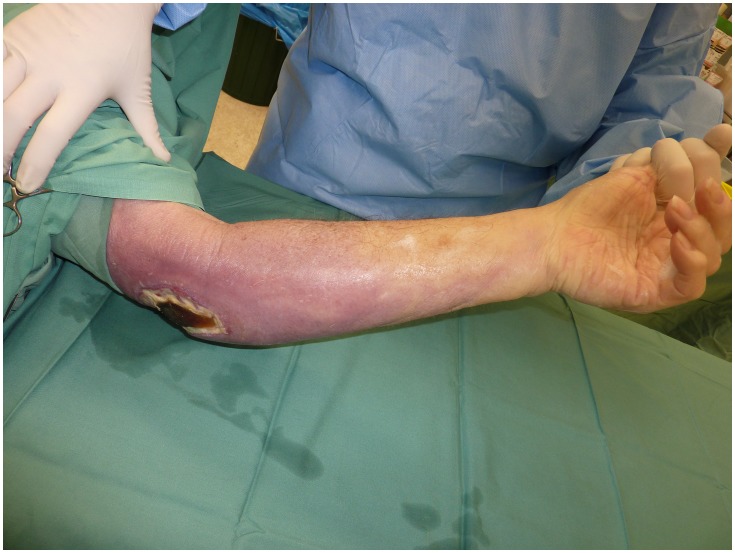
Oedematous *M. ulcerans* lesion of left elbow region showing necrosis.

The median age at diagnosis for those with oedematous lesions was 70 years (IQR 17–82 years) which was not significantly different to those with non-oedematous lesions [57 years (IQR 38–74); p = 0.51]. Twelve (71%) patients were male. Three patients had diabetes, 1 of whom also had carcinoma of the prostate. The median duration of symptoms prior to diagnosis was 20 days (IQR 15–33 days). This was significantly shorter than those with non-oedematous lesions [42 days, (IQR 28–73); p = 0.007]. Twelve (71%) lesions were located on the upper limb: 7 affecting the dorsum of the hand (figure 2), 4 the elbow (figure 3) and 1 the wrist/forearm. Five (29%) lesions were located on the lower limb: 3 affecting the ankle, 1 the toe, and 1 the knee. At diagnosis 4 (24%) lesions were classified as WHO category one, 6 (35%) as category two and seven (41%) as category three.

16 (94%) patients were initially diagnosed with cellulitis and received a median 14 days (IQR 9–17 days) of antibiotics for the cellulitis prior to the diagnosis of *M. ulcerans*. Ten (63%) were reported to have improved initially on antibiotic therapy. Patients received a median of 3 different antibiotics; those used (with the number and percent of patients where improvement was observed in parenthesis) were: oral cephalexin (6/10; 60%), intravenous (IV) cephazolin (8/9; 89%), IV or oral flucloxacillin/dixcloxacillin (6/9; 67%), oral amoxicillin/clavulanic acid (0/3; 0%), IV ceftriaxone (2/2;100%), IV vancomycin (1/2;50%), oral clindamycin (1/1; 100%), IV gentamicin (1/1; 100%) and oral doxycycline (1/1; 100%). Eleven (65%) patients required hospitalization for treatment of their ‘cellulitis’. One case was misdiagnosed as an inflammatory mass.

Fever was present in 6/12 (50%) cases in which temperatures were measured. If elevated, the median temperature was 38.5°C (range 37.9–39.7°C). Pain was present in 13/15 (87%) cases in which it was reported. The WCC was elevated in 7/13 (54%) cases in which it was available with a median WCC in these cases of 14.5×10^9^/l (range 13.2–18.8×10^9^/l). The neutrophil count was elevated in 8/13 (62%) cases with a median value for these cases of 11.2×10^9^/l (range 8.4–4.7×10^9^/l). The CRP was elevated in 9/12 (75%) cases with a median value in these cases of 91 mg/l(IQR 27–145 mg/l). Blood cultures were negative in 8/8 (100%) of cases in which they were collected. For the one case not misdiagnosed as cellulitis pain but not fever was reported, and the WCC, neutrophil count and CRP were within the normal range.

The diagnosis of *Mycobacterium ulcerans* was made via a swab specimen in 9 (52%) cases (if the lesion had ulcerated) and via a biopsy in 8 (47%) cases (when no ulceration present). When performed, PCR was positive in 16/16 (100%) cases, AFB smear was positive 10/12 (83%) cases and mycobacterial culture was positive in 5/10 (50%) cases.

The median duration of specific antibiotic treatment for oedematous lesions was 84 days (IQR 67–96); 13/17 (76%) had treatment for more than 60 days. The initial antibiotic regimen was rifampicin combined with ciprofloxacin in 11 (65%), clarithromycin in 5 (29%) and moxifloxacin in 1 (6%) case. Sixteen (94%) cases developed significant necrosis and required surgical debridement, 10 (59%) required a split skin graft and 1 also required a vascularised free flap. Prednisolone was used in 5 (29%) cases to treat secondary paradoxical reactions.

All patients healed their lesions on treatment and no recurrences were detected after 12 months of follow-up.

On multivariable analysis, there was an increased likelihood of a lesion being oedematous if it was located on the hand (OR 85.62, 95% CI 13.69–535.70; P<0.001), elbow (OR 7.83, 95% CI 1.39–43.96; p<0.001) or ankle (OR 7.92, 95% CI 1.28–49.16; p<0.001), or the patient had diabetes mellitus (OR 9.42, 95% CI 1.62–54.74; p = 0.02). (table 1) No association was found with sex or age.

**Table 1:** Logistic regression model showing adjusted and unadjusted associations between identified factors and oedematous *Mycobacterium ulcerans* lesions.

Variable	n (%) in cohort	n (%)* oedematous lesions	Unadjusted OR (95%)	p-value	Adjusted OR (95% CI)	p-value
Age						
<15	19 (8.0)	2 (10.5)	2.97 (0.50,17.50)	0.19	2.48 (0.26,23.50)	0.62
15–60	105 (44.1)	4 (3.8)	1		1	
>60	114 (47.9)	11 (9.7)	2.70 (0.83–8.75)		1.76 (0.45,6.88)	
Sex						
Female	116 (48.7)	5 (4.3)	1	0.09	1	0.51
Male	122 (51.3)	12 (9.8)	2.42 (0.83–7.10)		1.55 (0.42,5.63)	
Diabetes						
No	220 (92.4)	14 (6.4)	1	0.15	1	0.02
Yes	18 (7.6)	3 (16.7)	2.94 (0.76–11.4)		9.42 (1.62,54.74)	
Immunosuppression						
No	220 (3.3)	15 (6.8)	1	0.88	-	-
Yes	16 (6.7)	1 (5.9)	0.86 (0.11–6.92)		-	-
Lesion Position						
Hand	13 (5.5)	7 (53.9)	59.11 (12.18–286.83)	<0.001	85.62 (13.69–535.70)	<0.001
Elbow	34 (14.3)	4 (11.8)	6.76 (1.44–31.74)		7.83 (1.39–43.96)	
Ankle	36 (15.1)	3 (8.3)	4.61 (0.89–23.84)		7.92 (1.28–49.16)	
Other	155 (65.1)	3 (1.9)	1		1	
Joint						
No	144 (60.5)	8 (5.6)	1	0.24	-	-
Yes	94 (39.5)	9 (9.6)	1.80 (0.69–4.85)		-	-

Percentages represent the proportion of the variable examined from the whole cohort with that variable.

## Discussion

In an Australian population, our study suggests that oedematous lesions are usually initially misdiagnosed and treated as bacterial cellulitis. This is not surprising as the presenting symptoms, signs and investigations are consistent with cellulitis; fever and pain are usually present and investigations show leucocytosis, neutrophilia and a raised serum CRP. This is in contrast to other forms of *M. ulcerans* which are classically painless and not associated with systemic symptoms [7]. Furthermore, perhaps related to treatment of bacterial superinfection or a degree of early immune control of the disease, in the majority of cases health-care providers report initial clinical improvement on antibiotic therapy targeting cellulitis, although lesions later deteriorated. Treatment for cellulitis leads to a delay in diagnosis, with patients receiving a median of 2 weeks of non-*M. ulcerans* specific antibiotics, and usually results in hospitalization, thereby increasing the cost and complexity of treatment [8]. Nevertheless, the rapid progression of lesions, and the associated pain and rapid tissue necrosis likely results in the finding from our study that the time from onset of symptoms until diagnosis is significantly less in oedematous compared to non-oedematous lesions.

Our study confirms previous findings that oedematous forms of *M. ulcerans* disease are less common [3]–[6] representing only 7% of cases over the 15 year study period. The reasons lesions become oedematous are unknown, but may relate to the virulence of the infecting organism, host characteristics including their general and local immune function, or perhaps the size and depth of the inoculum [7]. We found that oedematous lesions were strongly associated with certain positions on the body, with the dorsum of the hands being affected 85 times more often, and the elbows and ankles being affected 8 times more often than other parts of the body. Reasons for this are not clear, but may include lower skin and subcutaneous temperatures [7], reduced local immune function or less subcutaneous tissue in these regions that may increase the ability of the organism to spread.

In comparison, a study of African patients reported 50% of oedematous lesions occurring on the upper limb, 38% on the lower limb and 12% on the torso compared with 79%, 21% and 0% in our study [5]. Of note patients in the African study had a higher proportion of more advanced lesions (79% versus 41% with WHO category 3 lesions), although patients in the African cohort had a younger median age at diagnosis (11 years compared with 70 years). As time to presentation for oedematous lesions was not reported in the African study it was not possible to assess its impact on the proportion of advanced lesions between the study settings [5].

Oedematous lesions can be extensive, rapidly progressive and lead to significant tissue loss as evidenced by 76% of lesions in our study presenting as WHO Category 2 or 3 lesions [1], [4], [5]. They are characterized by the presence of large numbers of extracellular AFBs. This suggests reduced organism inhibition by the host's immune system, either through organism or host factors. This is supported in our study by the association of an increased likelihood of oedematous lesions in patients with diabetes, likely related to its associated immunosuppressive effects [10]. Conversely however, we did not find an association between oedematous lesions and patients who were immunosuppressed.

The diagnosis of oedematous forms of MU can be hampered if there is no ulceration as swabs of the skin surface for PCR and AFB staining are often negative [5] and can lead to false negative diagnoses. It is therefore important for clinicians to recognize that other diagnostic methods are required; either a biopsy of the lesion including subcutaneous tissue, or aspiration of the lesion which may avoid ulceration of lesions that can occur at the biopsy site [11].

Despite the use of appropriate antibiotics, in our experience oedematous lesions usually ulcerate, often well into antibiotic treatment, and require debridement +/− skin grafting. The cause for the ulceration on treatment is not clear but may result from tissue swelling secondary to paradoxical reactions, which are more common with antibiotic treatment of oedematous forms of *M. ulcerans* [12], and may lead to ischaemia of skin and superficial tissues. Another cause may be a delayed effect of persisting mycolactone toxin [13]. It will be important to try and prevent this late tissue loss to maximize the benefits of early treatment by preserving involved tissue and more research needs to be performed in this area. In our experience potential treatments include the use of prednisolone to reduce the amount of tissue swelling associated with paradoxical reactions [14], or subcutaneous tissue debridement to reduce the burden of necrotic and inflamed tissue and potentially mycolactone toxin.

There are some potential limitations to this study. Firstly, due to its observational design there may be other unmeasured confounders not taken into account in the analysis of associations with oedematous *M. ulcerans* lesions that could potentially affect the validity of the findings. Observed associations should be further studied in prospective trials. Secondly, these results are from an Australian cohort and may not be generalisable to populations elsewhere.

### Conclusions

In an Australian population, oedematous *M. ulcerans* lesions present with similar symptoms, signs and investigation results to, and are commonly misdiagnosed as, bacterial limb cellulitis leading to delays in their diagnosis and treatment. In patients exposed to areas endemic for *M. ulcerans*, it is important for clinicians to have a high index of suspicion of *M. ulcerans* in cases presenting as cellulitis, especially if affecting the hand, elbow or ankle, occurring in patients with diabetes, or where an adequate response to routine antibiotics used for cellulitis has not been achieved.

## Supporting Information

Checklist S1 STROBE checklist.
(DOC)
